# A Rare Case of Oral Papillomatosis in a Goat Kid

**DOI:** 10.1155/2022/1598256

**Published:** 2022-05-05

**Authors:** Mohammed Naji Ahmed Odhah, Bashiru Garba, Chan Xin Wen, Mohd Farhan Hanif Reduan

**Affiliations:** ^1^Department of Veterinary Clinical Studies, Faculty of Veterinary Medicine, University Malaysia Kelantan (UMK), Kampus Kota, Locked Bag 36, Pengkalan Chepa, 16100 Kota Bharu, Kelantan, Malaysia; ^2^Department of Veterinary Public Health, Faculty of Veterinary Medicine, Thamar University, 87246 Dhamar, Yemen; ^3^Department of Veterinary Public Health and Preventive Medicine, Faculty of Veterinary Medicine, Usmanu Danfodiyo University, Sokoto, Nigeria; ^4^Department of Paraclinical Studies, Faculty of Veterinary Medicine, Universiti Malaysia Kelantan, Pengkalan Chepa, 16100 Kota Bharu, Kelantan, Malaysia

## Abstract

The clinical management and outcome of a case of caprine papillomatosis in a 2-week-old kid goat was reported. Based on the PCR result, contagious ecthyma (CE) was ruled out. Based on the history and physical examination findings, the case was diagnosed as severe clinical case of papillomatosis in a goat's kid. The treatment procedure was administrated with flunixin meglumine (5%) 1.1 mg/kg, 0.5 ml, IM, SID, 3 days, and Penstrep (procaine penicillin, dihydrostreptomycin sulphate) 2 ml and a topical preparation for 5 days to prevent secondary bacterial infection. Also, Vitavet (multivitamin–vitamins A, D3, and B12), 1 ml/10 kg, 1 ml, IM, SID, was administered for 3 days to boost the immune system.

## 1. Introduction

Papillomatosis in goats is a neoplastic contagious disease, characterized by the presence of multiple papillomas that appear as exophytic proliferation giving rise to papillary lesions with finger-like projections [1]. These lesions are known to either spontaneously regress or progress to malignancies, leading to severe outcomes like upper alimentary tract and urinary bladder carcinomas [2]. Caprine papillomatosis is caused by a globally disseminated papilloma virus species following direct contact with an infected animal via abraded skin or other mucosal lesions, as well as fomites such as contaminated feed troughs, ear tagging pliers, and rectal palpation sleeves. The three common types of goat papillomas are mammary, cutaneous (excluding the mammary gland), and genital [3].

Although the actual economic burden of caprine papilloma virus infection has not been ascertained, infection is predominantly reported in adult goats and frequently giving rise to feeding and breathing difficulties, weight loss, and growth retardation [2]. Similarly, goat papillomatosis can result in the suppression of the immune system giving opportunities to secondary bacterial infections, mainly in the oral cavity, teats, and udder which are characterized by pain, inappetence, and mastitis.

Here we present a rare case of oral papillomatosis in a two-week-old Boer goat kid presented to the Veterinary Teaching Hospital, Faculty of Veterinary Medicine, Universiti Malaysia Kelantan.

## 2. Case Presentation

This is a case of a 2-week-old female Boer goat breed with a body weight of 5 kg which is managed under a semi-intensive system along with three other goats. The owner noticed that the goat kid was having a mass on the lower lip a week after being kidded and unable to suckle. In addition, the client also noticed bilateral facial swelling and frothy saliva drooling from its mouth. The vaccination and deworming status were not up to date.

Upon physical examination, the goat kid appeared, weak but responsive. Clinical examination showed it was pyrexic with mild tachycardia. The mucus membrane was pale (pinkish) with a capillary refill time of less than two seconds, and there was enlargement of both submandibular lymph nodes. The dehydration status was less than five percent. The kid also appeared weak and lethargic. The body condition score was 2/5, and the FAMACHA score was 2/5.

**Figure 1:**
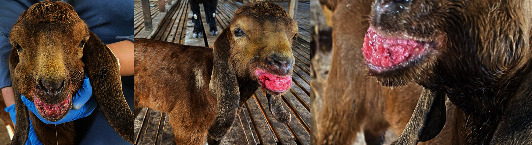
Papillomavirus lesions in the kid showing a mass on the lower lip, buccal mucosa, hard and soft palates, cauliflower-like appearance.

Figure 1 shows the kid on presentation with an approximately 3 cm × 3 cm pinkish, irregular, and cauliflower-like appearance, sessile mass on the lower lip, buccal mucosa, and hard and soft palates.

### 2.1. Diagnostic Approach

A biopsy on the fresh mass was taken from the oral mass tissue and fixed in a 10% formalin and sent for a histopathological analysis, following the procedure reported by [4].

**Figure 2:**
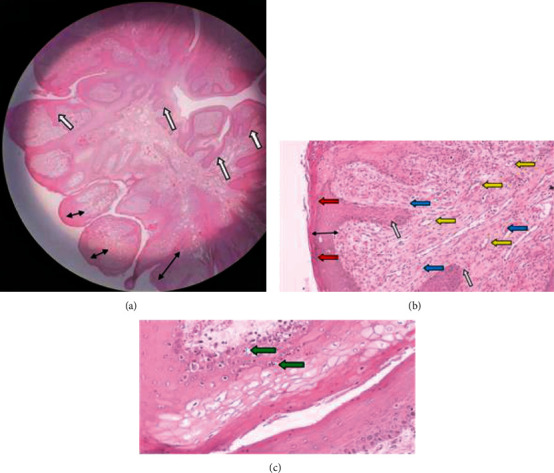
The histopathology result in this case of caprine papilloma tissues. (a) Overall hyperproliferation on the epidermis layer, characterized by thickening of the epidermal layer (black, double head arrow) and formation of rete peg which from epidermis layer extended to dermis layer (white arrow), indicating acanthosis. (b) Presence of nucleated cells within the stratum corneum (red arrow), indicating parakeratotic hyperkeratosis. Evidence of angiogenesis with the presence of the luminal structure surrounded by the endothelial cell (yellow arrow) and filled with some red blood cells. Hypercellularity of the dermis layer, filled with spindle-shaped fibroblast (blue arrow). (c) Presence of ballooning cell indicating koilocytosis (green arrow). H&E staining and magnification (a) 4x, (b) 10x, and (c) 40x.

The histopathological analysis of the oral tissue on the lowest magnification revealed an overall hyperproliferation of the epidermis layer of the oral cavity, characterized by thickening of the epidermal layer (black, double head arrow) and the formation of rete peg which from epidermis layer extended to dermis layer (white arrow), indicating acanthosis as shown in Figure 2(a).

Similarly, nucleated cells within the stratum corneum (red arrow) were observed indicating parakeratotic hyperkeratosis (Figure 2(b)). Moreover, evidence of angiogenesis was detected with the presence of the luminal structure surrounded by the endothelial cell (yellow arrow), filled with some red blood cells (hypercellularity of the dermis layer), with spindle-shaped fibroblast (blue arrow), were also observed (Figure 2(b)). On a high magnification (40x), there was also marked ballooning and koilocytosis which is a diagnostic marker for papillomavirus infection and can be observed morphologically in histological sections (Figure 2(c)) [5].

Molecular detection using the FAP59/FAP64 and MY09/MY11 primer pairs for the L1 gene region as well as PPP1 and PPP4 for parapoxvirus was conducted as earlier reported [6, 7]. The result came out positive for papillomavirus and negative for parapoxvirus (PPV) and contagious ecthyma (CE) according to the PCR result for positive and negative controls.

Similarly, bacterial culture of swab samples revealed no positive bacterial growth. Hence, fibromatous epulis was ruled out based on the topography of the mass, in which case epulis arises from the gingiva sulcus; however, the mass observed in the present case not only involved the lower mandible (on the lower incisor part) but can also be seen in the buccal mucosa, as well as the hard and soft palates. Furthermore, squamous cell carcinoma (SCC) was ruled out based on the age of the kid and the nature of the mass. Squamous cell carcinoma is a malignant tumour affecting the epidermal cells of adult goats in which the cells show differentiation to keratinocytes [8]. Hence, it is less likely for a young kid (2 weeks old) to develop SCC. Also, SCC in goat tends to be invasive and ulcerative. So, based on the various diagnostic tests analysed (PCR, histopathology, and culture), a confirmatory diagnosis of caprine papillomatosis was arrived at based on the histopathology and PCR results. Additionally, the prognosis for the case was thought to be good, meaning that with intervention in the form of antibiotic therapy and supportive care, the lesions will regress.

### 2.2. Management and Client Advice

The kid was given flunixin meglumine (5%) 1.1 mg/kg BW, 0.5 ml, IM, SID, 3 days, and penicillin-streptomycin (Penstrep-procaine penicillin, dihydrostreptomycin sulphate) 2 ml and a topical preparation of the penicillin-streptomycin for 5 days to prevent secondary bacterial infection. Also, Vitavet (multivitamin–vitamins A, D3, and B12) 1 ml/10 kg, 1 ml, IM, SID, was administered for 3 days to boost the immune system.

On the other hand, the client was advised and guided to disinfect the pens and equipment using 2-4% formaldehyde solution. He was also advised to provide nursing doe for the kids, wear gloves when handling the animals, sanitize all the equipment used for the infected animals, and maintain farm hygiene. Finally, he was advised to vaccinate the remaining goats from the same flock with the autogenous vaccine.

**Figure 3:**
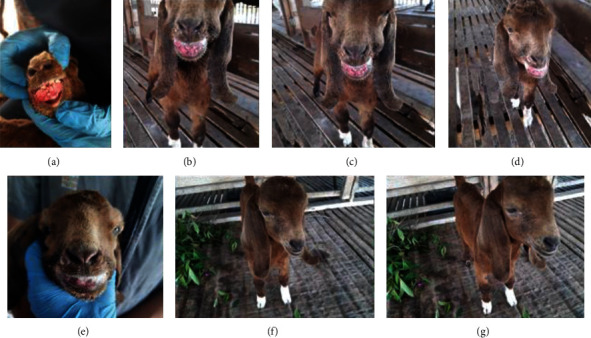
The progression of treatment after administration of flunixin meglumine, Penstrep (procaine penicillin, dihydrostreptomycin sulphate), and Vitavet (multivitamin–vitamins A, D3, and B12). There was marked regression of the oral mass fibrosis growth from (a) commencement of treatment to (g) final discharge.

Three weeks after the treatment commenced, the lesions were completely healed and the animal fully recovered (Figure 3).

## 3. Discussion

Papillomavirus infection has been described in many animal species and in humans, but it is rarely reported on goats. The disease is one of the most important diseases of large and small ruminants [9]. Caprine papillomatosis (papillomas and fibropapillomas) is a common viral disease of the oral cavity, lips, udder, head, and neck mostly of young goats, manifested as benign tumours or warts, caused by caprine papillomavirus (CPV) that has variety serotypes [3, 6]. Although the disease can occur in goats, it is most commonly seen among adult goats. Hence, the intrigue in this case is where the disease was reported in a two-week-old goat. Ironically, both the mother and all other members of the herd had no lesions indicative of the disease. Traditionally, when on clinical examination, the lesions are multiple and appear as fibromatous epulis and benign, noninvasive growth, predominated with periodontal ligament stroma (pedunculated and multiple), and it may be sufficient and characteristic enough to confirm the diagnosis as papillomatosis. However, a further test is needed to confirm the diagnosis.

The papilloma lesions can be found in the oral cavity in goats as well as the mammary gland which is more common. Depending on the degree of keratinization, the color of the surface of the lesion varies between red, pink, and white, with the most common places being the palate and the tongue. Histopathological examination of oral cavity lesions revealed similar pathology with acanthosis, keratinocyte multinucleation, and koilocytosis. These histological features are usually seen when the infection becomes productive, leading to squamous epithelial differentiation. Although these features can be mistaken for squamous cell carcinoma, the size of the lesion, the location, and the cauliflower-like appearance of papilloma can help distinguish them [10]. Fortunately, the polymerase chain reaction method which is recommended for accurate identification of the pathogen agent by amplifying the target gene was able to identify the virus from genomic DNA obtained from oral tissue biopsy. Earlier studies have demonstrated the potential of the PCR technique in identifying papillomavirus using primers FAP59 and FAP 64 targeting the conserved L1 gene fragment [7].

The virus can enter and infect the keratinocyte of the skin stratum basale. After that, the virus amplifies, leading the basal keratinocyte at stratum basale to undergo differentiation. This will lead to the expression of viral protein that can stimulate cell growth [11]. The hyperproliferation caused the formation of the exophytic mass, which is warts as a consequence of the acanthosis [2]. The exophytic multiple, cauliflower-growths (warts) are found on the lower lip, buccal mucosa, and hard and soft palates [12]. Papillomas can be painful and in most cases require a course of antibiotic treatment to prevent complications as a result of secondary bacterial infections. When goats have many growths that eating becomes problematic, surgical excision is recommended. Although most cases of caprine oral papillomas regress on their own, multiple other new treatment options have been reported, including autohemotherapy and autogenous vaccine. An earlier study in Erode District of Tamilnadu reports that autohemotherapy was found to be the most effective therapy to cure papillomatosis [9]. However, in this case, only topical and systemic antibiotic therapy was given as well as supportive care in the form of vitamins and rest.

## 4. Conclusion

In conclusion, the present study revealed that management of goat kid papillomatosis can be successful by providing adequate supportive care, in addition to appropriate treatment with suitable local and systemically antibiotics, vitamins, and flunixin meglumine within four weeks.

## Data Availability

Data associated with this report are available in the published article.
